# Graphene surface plasmon sensor for ultra-low-level SARS-CoV-2 detection

**DOI:** 10.1371/journal.pone.0284812

**Published:** 2023-04-25

**Authors:** Md. Mahbub Hossain, Muhammad Anisuzzaman Talukder

**Affiliations:** Department of Electrical and Electronic Engineering, Bangladesh University of Engineering and Technology, Dhaka, Bangladesh; Wayamba University of Sri Lanka, SRI LANKA

## Abstract

Precisely detecting the ultra-low-level severe acute respiratory syndrome coronavirus 2 (SARS-CoV-2) is crucial. The detection mechanism must be sensitive, low-cost, portable, fast, and easy to operate to tackle coronavirus disease 19 (COVID-19). This work proposes a sensor exploiting graphene surface plasmon resonance to detect SARS-CoV-2. The graphene layer functionalized with angiotensin-converting enzyme 2 (ACE2) antibodies will help efficient adsorption of the SARS-CoV-2. In addition to the graphene layer, ultra-thin layers of novel two-dimensional materials tungsten disulfide (WS_2_), potassium niobate (KNbO_3_), and black phosphorus (BP) or blue phosphorus (BlueP) used in the proposed sensor will increase the light absorption to detect an ultra-low SARS-CoV-2 concentration. The analysis presented in this work shows that the proposed sensor will detect SARS-CoV-2 as small as ∼1 fM. The proposed sensor also offers a minimum sensitivity of 201 degrees/RIU, a figure-of-merit of 140 RIU^−1^, and enhanced binding kinetics of the SARS-CoV-2 to the sensor surface.

## 1 Introduction

Severe acute respiratory syndrome coronavirus 2 (SARS-CoV-2) is a critical biological pathogen responsible for coronavirus disease 2019 (COVID-19). Recently, COVID-19 has caused an unprecedented health problem worldwide due to the high progression rate of fatality. SARS-CoV-2 is a positive-sense single-stranded ribonucleic acid (RNA) virus [1]. Several research groups have proposed and demonstrated efficient, cost-effective, and real-time detection techniques for the SARS-CoV-2 virus [2–8]. Culture-based techniques that detect nucleic acid or proteins and serological-based techniques that detect the created antibodies are commonly used for virus diagnosis [9]. Recent advances in molecular technology have led to the development of nucleic acid-dependent amplification techniques for virus detection, e.g., the reverse transcriptase quantitative polymerase chain reaction (RT-qPCR) technique. RT-qPCR is known as the gold standard for detecting several critical viruses, such as SARS-CoV-2, human immunodeficiency virus (HIV), and cytomegalovirus (CMV) [2].

RT-qPCR is highly sensitive and selective for SARS-CoV-2 detection [3]. Nevertheless, its application is limited due to significant false-negative cases (∼15%), long processing time, costly instruments, and the requirement of skilled human resources and uninterrupted power supply over a long period [4, 5]. Conversely, colorimetric-based loop-mediated isothermal amplification (LAMP) techniques have been proposed for SARS-CoV-2 detection to overcome the limitations of an RT-qPCR technique [5]. LAMP techniques show a high nucleic-acid amplification efficiency but are time-consuming and tedious as they use electrophoresis for detection. Additionally, the decision regarding the color change of reaction vessels by human eyes is potentially a subjective issue and may significantly impact the test results [6]. A chest computer tomography (CT) scan can also be used to detect the SARS-CoV-2 [7]. However, such a technique cannot be used for asymptomatic patients, early-stage detection, and the measurement of the mass density of virus [10]. Serological tests, e.g., enzyme-linked immunosorbent assay (ELISA), can also be used for SARS-CoV-2 diagnosis [8]. Serological methods have shown high efficiency and are low-cost, but they suffer from low sensitivity (*S*) and false-negative reports [3].

In the last few years, optical sensors based on plasmonics have attracted significant attention in virus detection due to their simplicity, flexibility, label-free operation, and short response time [2, 11–15]. Plasmonics-based optical sensors have been proposed to detect many critical pathogens, such as dengue virus envelope (E)-protein, thyroglobulin, HIV-1, and SARS-CoV-2 [3, 16–20]. Besides, optical biosensors have recently been proposed to detect SARS-CoV-2 proteins, such as spike and nucleo-capsid [21–25]. In particular, a toroidal plasmonic meta-sensor has recently been proposed to detect SARS-CoV-2 spike (S)-protein using terahertz (THz) wavelength signal, demonstrating a limit of detection (LoD) of only ∼4.2 fM [19]. However, the plasmonic meta-sensor suffers from insensitivity to S-protein concentration between 20 and 50 fM and low quality-factor (Q-factor).

More recently, a surface plasmon resonance (SPR) based method has been proposed to examine the affinity of SARS-CoV-2 S-protein to angiotensin-converting enzyme 2 (ACE2) [26]. Additionally, plasmonic photo-thermal effect and localized SPR (LSPR) have been proposed to detect selected sequences of SARS-CoV-2 by nucleic acid hybridization techniques [4]. However, thermo-plasmonic heat cannot discriminate between two similar gene sequences, and a sensor employing this effect shows low detection accuracy. Furthermore, a near-infrared (NIR) plasmonic sensor has been suggested for SARS-CoV-2 S-protein detection using a phase interrogation technique [3]. Although an NIR plasmonic sensor shows high sensitivity, its performance is limited due to the complex technology required for phase variation measurement.

Recently, graphene surface plasmon (SP) has drawn significant interest for application in sensing due to two-dimensional (2-D) graphene’s promising properties, such as high *π*-conjugation structure, shallow thickness and mass, and high mechanical strength [27]. In SPR sensors, the sample bio-molecules should be efficiently adsorbed by the sensor surface to increase the sensitivity [28–30]. Therefore, bio-molecular recognition elements (BREs) are often placed on top of SPR-based sensors to functionalize the metal film for enhanced bio-molecule adsorption. Since graphene surfaces can be modified by introducing different BRE functional groups, such as epoxy, hydroxyl, ketone, and carboxyl in their basal plane, graphene-based sensors show high bio-molecule adsorption capability [31]. Recently, apart from graphene, a few other 2-D materials, such as molybdenum disulfide (MoS_2_) and blue phosphorus (BlueP), have been shown to significantly improve sensor sensitivity when used in simple metal-based SPR sensors [32]. Moreover, an SPR biosensor based on bimetallic films gold (Au)-silver (Ag) and BlueP has shown good sensitivity [33] but limited performance in detecting ultra-low concentrations of biological molecules. However, using different 2-D materials like tungsten disulfide (WS_2_), potassium niobate (KNbO_3_), and black phosphorus (BP) in addition to graphene in a silver (Ag) based SPR biosensor is still unexplored, although they have optical properties promising for an SPR sensor.

This work proposes a graphene SPR sensor for ultra-low-level SARS-CoV-2 detection. SPR plays a pivotal role in the sensing principle of the proposed sensor [34–36]. The proposed sensor is based on the Kretschmann configuration, thus being simple. ACE2 functionalizes the graphene layer for efficient adsorption of the SARS-CoV-2 S-protein sample [20]. The proposed sensor uses thin layers of novel 2-D materials between graphene and Ag layers, such as WS_2_, KNbO_3_, and BP or BlueP, to increase the light absorption and hence, the sensor’s sensitivity. The optical and electronic properties of 2-D hetero-structures highly depend on the number of 2-D material layers and the stacking patterns. Due to their excellent sensitivity enhancement effects, such as 2-D materials BlueP/BP, KNbO_3_, and WS_2_, we believe our proposed biosensor will find applications in practical biosensing [37].

This work is a theoretical work based on detailed analytical and numerical calculations. We apply the finite difference time domain (FDTD) simulation technique to characterize the sensor response to incident light and determine the sensor performance parameters. SARS-CoV-2 S-proteins are detected by calculating the change in resonance angle for SPR excitation. We use the Langmuir model to calculate the equilibrium dissociation constant (*K*_*D*_) to determine the binding kinetics between ACE2 and S-protein [16]. The proposed sensor shows the prospect of detecting ultra-low SARS-CoV-2 concentration of only ∼1 fM, which is crucial for the early detection of this deadly virus. The proposed graphene SPR sensor also shows a high sensitivity, figure-of-merit (FoM), selectivity, and resolution while detecting the SARS-CoV-2 compared to the state-of-the-art SPR sensors. Furthermore, the proposed sensor offers a significantly small *K*_*D*_, showing enhanced binding of SARS-CoV-2 on the sensor surface.

The rest of the paper is prepared as follows: Sec. 2 illustrates and discusses the proposed sensor configuration and optimization of layer thicknesses. Then, Sec. 3 presents the optical properties of different materials, theoretical analysis of sensor performance parameters, and simulation methods. Next, we present and discuss the SARS-CoV-2 detection approach, analysis of binding between ACE2 and S-protein, and the calculated sensor performances in Sec. 4. Finally, in Sec. 5, we conclude the proposed sensor results.

## 2 Proposed sensor

### 2.1 Configuration

**Fig 1:**
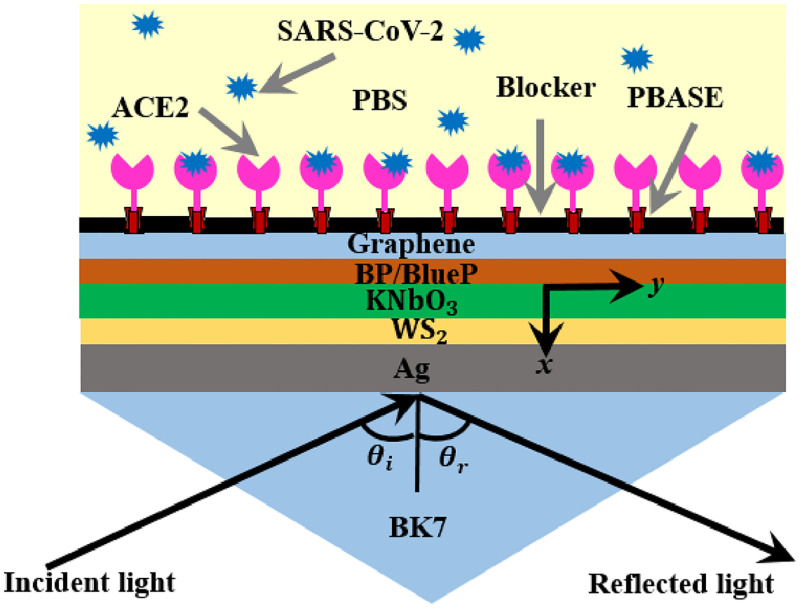
Schematic illustration of the proposed graphene SPR sensor for SARS-CoV-2 S-protein detection.

The proposed graphene SPR sensor is designed based on the Kretschmann configuration, as shown in Fig 1. The incident light on the metal–dielectric interface at the resonance angle (*θ*_*r*_) excites SPR, significantly absorbing the incident light. The *θ*_*r*_ for SPR changes based on the refractive index of the dielectric material, i.e., the sample layer. The sample layer refractive index varies due to the presence of SARS-CoV-2. Therefore, SARS-CoV-2 can be detected by measuring the change in *θ*_*r*_. SARS-CoV-2 samples will be placed on the top surface of the proposed sensor structure, which is a graphene layer. In practice, we need binding molecules to immobilize antibodies on the graphene surface to capture SARS-CoV-2. We use 1-pyrenebutyric acid N-hydroxy-succinimide ester (PBASE) that permits the binding of functional groups to graphene without disrupting the carbon atomic structure [38] and acts as an interfacing molecule and a probe linker [20]. PBASE contains an aromatic pyrenyl group, which physically interacts with graphene through *π*–*π* interaction. PBASE also contains a succinimidyl ester group, which covalently reacts with the amino group on the antibody by an amide bond [39].

SARS-CoV-2 consists of four fundamental physical proteins: S, E, matrix, and nuclei-capsid proteins. S-protein is immunogenic and shows amino acid sequence variation, permitting the specific detection of SARS-CoV-2 [40]. Hence, this work uses S-protein as the sensing element to identify SARS-CoV-2 [20]. The S-protein contains protrusions that only bind to specific receptors on the host cell, such as ACE2, dipeptidyl peptides-4, amino-peptides N, and carcinoembryonic antigen-related cell adhesion molecule 1 [41]. Recent research results have confirmed that ACE2 is an effective receptor for SARS-CoV-2 S-protein, with SARS-CoV-2 grasping ACE2 cells primarily by endocytosis [42]. Therefore, thiol-tethered DNA is used in this work as an ACE2 layer for receiving and detecting SARS-CoV-2 samples [43].

In this work, ACE2 antibodies are placed throughout the top surface of the sensor with 50 nm separations between the neighboring ACE2 antibodies. ACE2 height and width are assumed to be 3 nm and 2.1 nm, respectively [17]. To block the free space between ACE2 antibodies, we use ethanolamine as a blocker [44]. Each ethanolamine blocker is 3.5 nm long and separated from neighboring blockers by 50 nm. The ethanolamine blockers support keeping the ACE2 antibodies static in their places and prevent the adsorption of non-specific elements on the graphene surface [45]. SARS-CoV-2 samples can be collected from human nasopharyngeal swabs and preserved in a phosphate-buffered saline (PBS) solution [46]. The PBS solution containing SARS-CoV-2 S-proteins makes the sensing layer, which can flow over the sensor surface through a flow channel as an analyte [45]. In this work, the PBS sensing layer volume is set to 100 *μ*L and 200 *μ*L to investigate the sensor performances. We note that the PBS is neutral to the SARS-CoV-2 S-protein and often used for analyzing proteins [47].

The proposed graphene SPR sensor is built on a semi-infinite boro-silicate (BK7) prism material, as shown in Fig 1. The light is incident on the multi-layer structure from the prism side, and the reflected light is recorded on the same side. The incident light excites surface plasmon polaritons (SPPs) at the metal–dielectric, i.e., metal–multi-layer interface. The excitation of plasmonic modes is sensitive to the thickness of the metal layer. This work uses a 46-nm-thick Ag layer as this thickness peaks the SPP excitation [48]. For SPR, Ag is preferred to other metals, such as Au or copper (Cu), as it shows dense plasmonic interaction with light at low loss [49]. The scattering cross-section of Ag is greater than other metal choices [50]. Besides, Ag offers a narrower SPR spectrum than other metals, which is essential for plasmonic biosensors.

The multi-layer 2-D structure interfaces with the metal layer with WS_2_. To date, MoS_2_ has been commonly used for such planar plasmonic structures. MoS_2_ and WS_2_ belong to the same family of chemical characteristics. However, WS_2_ is more stable than MoS_2_, especially at high temperatures [51]. Additionally, WS_2_ effectively absorbs more light than MoS_2_ or other transition metal dichalcogenide (TMD) materials [52, 53]. Therefore, WS_2_ helps decrease the incident light’s reflection when interfaced with Ag.

In the proposed structure, a KNbO_3_ layer follows the WS_2_ layer. KNbO_3_ has a high optical permittivity that enhances the electric flux density within the sensor [54]. Additionally, the imaginary part of the refractive index of KNbO_3_ is zero. Hence, KNbO_3_ increases the light confinement without incurring losses. The layer that follows KNbO_3_ is a phosphorene family material BP or BlueP. The BP or BlueP layer is sandwiched between KNbO_3_ and graphene. The sensitivity of the proposed sensor increases significantly as BP and BlueP have a high real part of the refractive index, enhancing the light confinement [55].

Using several 2-D materials in the proposed structure will increase the fabrication complexity slightly. However, the fabrication of various 2-D materials is usually cheap nowadays. In addition, the significant performance enhancement from the proposed sensor justifies using different 2-D materials at a reasonable cost increase. Furthermore, the sensor will be available for reuse after the purification of the used sensing channel.

### 2.2 Optimization of layer thicknesses

The proposed graphene SPR sensor has several layers, each having an essential effect on the overall performance. However, appropriate optimization of layer thicknesses is critical to get the best response from the proposed sensor. Here, we have optimized the layer thicknesses of the proposed sensor using the approach discussed in Refs. [56, 57]. In particular, we examine the effect of each layer thickness on the reflected light intensity (*R*) profile as a function of the incidence angle (*θ*_*i*_). The optimization of layer thicknesses depends on the minimum reflected light intensity (*R*_min_) and full-width at half-maximum (FWHM) of the *R*-profile. The FWHM is the spectral width of the *R*-profile corresponding to 50% reflectivity (FWHM = Δ*θ*_*i*,(0.5)_) [58]. While the light absorption is maximum on the sensor surface at *R*_min_, the FWHM represents the loss in the metal layer. Therefore, both *R*_min_ and FWHM are crucial for the sensitivity enhancement of a sensor, and an optimized layer thickness should produce both *R*_min_ and FWHM as small as possible.

**Fig 2:**
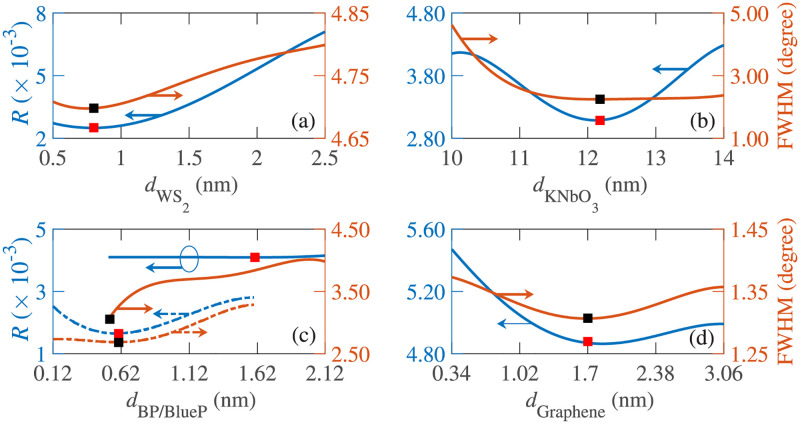
Reflectance (*R*) of the proposed sensor structure in Kretschmann configuration against layer thicknesses of (a) WS_2_, (b) KNbO_3_, (c) BP (solid) and BlueP (dashed), and (d) Graphene.

The layer thicknesses are optimized sequentially. First, the layer thickness of WS_2_ is optimized, and then that of KNBO_3_, BP, and graphene. To optimize the layer thicknesses, we change each layer thickness while the thicknesses of all other layers are fixed. We optimize the WS_2_ layer thickness from calculations, as shown in Fig 2(a). When WS_2_ = 0.8 nm, *R*_min_ and FWHM show minimum values. Furthermore, the increase of the WS_2_ thickness ($d_{{\text{WS}}_{2}}$) broadens the FWHM of *R*-profiles as *R*_min_ value increases. Therefore, we set $d_{{\text{WS}}_{2}}=0.8$ nm for the proposed sensor structure. Subsequently, we calculate the effects of KNbO_3_ keeping $d_{{\text{WS}}_{2}}$ at the optimized value. We determine *R*_min_ and FWHM values when the thickness of KNbO_3_ ($d_{{\text{KNbO}}_{3}}$) is varied from 10 nm to 14 nm, as shown in Fig 2(b). In this case, both *R*_min_ and FWHM are minimum when $d_{{\text{KNbO}}_{3}}=12.2$ nm.

Following a similar procedure, we optimize *d*_BP_ and *d*_BlueP_ when *d*_Ag_, $d_{{\text{WS}}_{2}}$, and $d_{{\text{KNbO}}_{3}}$ are at their optimized values. We show the change in *R* and FWHM with *d*_BP_ and *d*_BlueP_ in Fig 2(c). We note that, initially, *R* decreases very minutely with *d*_BP_ and is minimum at *d*_BP_ = 1.59 nm. However, *R* increases when *d*_BP_ > 1.59 nm. By contrast, FWHM always increases with *d*_BP_ and is minimum when *d*_BP_ = 0.53 nm, which is BP mono-layer thickness. We find that when *d*_BP_ increases from 0.53 nm to 1.59 nm, *R*_min_ decreases by 0.52% whereas FWHM increases by 16.66%. A narrow FWHM of the *R*-profile is required for high signal-to-noise ratio (SNR) and accuracy of *θ*_*r*_ detection [59]. Therefore, *d*_BP_ = 0.53 nm is set for the proposed structure. We note that *R* decreases initially with *d*_BlueP_ and is minimum at *d*_BlueP_ = 0.615 nm. However, *R* increases when *d*_BlueP_ > 0.615 nm. The thickness of a BlueP single layer is 0.123 nm [60]. Therefore, 0.615 nm represents five layers of BlueP. On the other hand, FWHM decreases slightly and becomes minimum at *d*_BlueP_ = 0.615 nm. Therefore, we set *d*_BlueP_ = 0.615 nm for the proposed structure.

The change of graphene layer thickness changes the surface plasmon wave vector, eventually changing *θ*_*r*_. Fig 2(d) shows that, as *d*_Graphene_ increases, both *R* and FWHM decrease since the incident light confinement at the metal–dielectric interface enhances [58]. When *d*_Graphene_ increases from mono-layer, i.e., 0.34 nm, to five layers, i.e., 1.70 nm, *R* decreases by 12.93% as the absorption of incident light increases. However, *R*_min_ and FWHM both increase when *d*_Graphene_ >1.70 nm [58]. Therefore, the proposed sensor uses *d*_Graphene_ = 1.70 nm.

## 3 Modeling and simulation

### 3.1 Optical properties

The optical properties of the proposed sensor’s layer materials are dispersive. Therefore, the sensor’s response depends on the incident light’s wavelength. The sensor has been designed for an incident wavelength of 633 nm, frequently used in experiments [67]. This work calculates the wavelength-dependent refractive index of the BK7 prism following the discussion presented in Ref. [58] and of Ag using the Drude-Lorentz model [61].

The wavelength-dependent refractive index of WS_2_ has been calculated using [62]

$$\begin{array}{c} n_{{\text{WS}}_{2}}=\sqrt{\frac{1}{2}[N+\frac{\delta }{\alpha }]\pm \frac{1}{2}\sqrt{{\left[N+\frac{\delta }{\alpha }\right]}^{2}-4n_{0}^{2}n_{\text{sub}}^{2}}}, \end{array}$$ (1)

where $N=n_{0}^{2}+n_{\text{sub}}^{2}$, *n*_0_ and *n*_sub_ are the refractive indices of air and substrate, respectively. The parameter *δ* is the fractional change of the complex reflection ratio, and *α* is defined as

$$\begin{array}{c} \alpha =4ik_{0}\;d_{{\text{WS}}_{2}}\;\frac{n_{0}n_{\text{sub}}^{2}\text{cos}\;\theta_{i}{\text{sin}}^{2}\theta_{i}}{\left(n_{0}^{2}-n_{\text{sub}}^{2})[n_{\text{sub}}^{2}-n_{0}^{2}+N\;\text{cos}\left(2\theta_{i}\right)\right]}, \end{array}$$ (2)

where *k*_0_ = 2*π*/λ and λ is the operating wavelength. The refractive index of KNbO_3_ depends on λ according to the following expression [63]

$$\begin{array}{c} n_{{\text{KNbO}}_{3}}=\sqrt{4.4222+\frac{0.09972}{\lambda^{2}-0.05496}-0.01976\lambda^{2}}. \end{array}$$ (3)

We have used the refractive index of BP from Ref. [64]. Furthermore, the refractive index of graphene has been calculated by [66]

$$\begin{array}{c} n_{\text{Graphene}}=3+i\frac{C_{G}}{3}\lambda , \end{array}$$ (4)

where *C*_*G*_ is a constant with a value of 5.44 *μ*m^−1^ [68]. The calculated refractive indices for BK7, Ag, WS_2_, KNbO_3_, BP, and graphene are given in Table 1. The indices of PBASE, ACE2, blocker, and PBS solution of the proposed sensor are obtained from the literature and are also given in Table 1.

**Table 1:** Refractive indices and thicknesses of the proposed sensor layers.

Material	Refractive index	Thickness (nm)	Reference
ACE2	1.13	2.10	[43]
Blocker	1.4539	3.23	[44]
PBASE	1.74	1.13	[45]
Sensing layer	1.3348	100	[47]
BK7	1.515	semi-infinite	[58]
Ag	Real: 0.055	46	[61]
	Imag: 4.285		
WS_2_	Real: 4.90	0.8	[62]
	Imag: 0.3124		
KNbO_3_	2.165	12.20	[63]
BP	Real: 3.50	0.53	[64]
	Imag: 0.01		
BlueP	Real: 2.1666	0.615	[65]
	Imag: 0.1005		
Graphene	Real: 3.0	1.70	[66]
	Imag: 1.1419		

The index of the sensing layer (*n*_*s*_) varies according to the following expression when SARS-CoV-2 S-proteins bind to ACE2 antibodies in the PBS layer [69]

$$\begin{array}{c} n_{s}=n+\beta D, \end{array}$$ (5)

where *n* is the index of the PBS solution, *β* is the index progress coefficient with a value of ∼0.186 cm^3^/gm for PBS [70, 71], and *D* is the mass density of S-protein in gram per deciliter. We calculate *D* using the following expression [48]

$$\begin{array}{c} D=C\times M, \end{array}$$ (6)

where *C* is the S-protein molar concentration in the PBS solution, and *M* is the S-protein molecular weight, which is 180 kDa or 180×2.5875×10^−19^ gm [42, 72]. We can write 1 fM = 1 × 10^−15^ gm × 411.04 gm/L, or = 4.1104 × 10^−12^ gm/dL. Also, 1 fM dissolved in 100 *μ*L PBS solution is equivalent to 4.1104 × 10^−12^ × 100 × 10^−6^ gm/dL. Therefore, *D* = 4.1104 × 10^−12^ × 100 × 10^−6^/10^−15^ gm/ dL ≃0.041104 gm/dL for 100 *μ*L PBS solution. Then *n*_*s*_ = 0.041104 × 0.00186 + 1.3348 ≃1.33485 for 1 fM S-protein concentration in 100 *μ*L PBS saline.

A similar procedure is applied for the 200-*μ*L PBS solution to determine *n*_*s*_ for varying S-protein concentrations.

**Fig 3:**
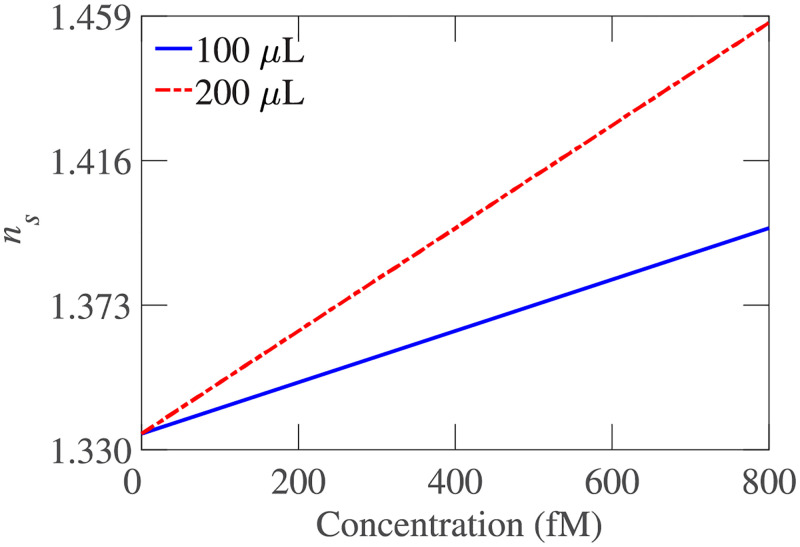
Sensing layer refractive index (*n*_*s*_) vs. SARS-CoV-2 S-protein concentration for 100-*μ*L and 200-*μ*L PBS solutions.

In this work, we consider 100-*μ*L and 200-*μ*L PBS in the sensing layer separately, where the molar mass of PBS is 411.04 gm/L. We vary the SARS-CoV-2 S-protein concentration from zero to 800 fM in the PBS solution. The sensing layer refractive index *n*_*s*_, due to the inclusion of the SARS-CoV-2 S-protein, is calculated using Eqs (5) and (6) and presented in Fig 3. When the S-protein concentration is zero, the refractive index of the sensing layer is 1.3348, which increases linearly as the S-protein concentration increases. We note that 200-*μ*L PBS shows a higher *n*_*s*_ than 100-*μ*L PBS as the mass density of S-protein increases with the PBS solution volume.

### 3.2 Sensor performance parameters

The sensitivity (*S*) and FoM are the main performance parameters of SPR-based sensors. These parameters are determined using the *R*-profile. The sensitivity is defined as the ratio of Δ*θ*_*r*_ and Δ*n*_*s*_ [73]

$$\begin{array}{c} S=\frac{\Delta \theta_{r}}{\Delta n_{s}}, \end{array}$$ (7)

where Δ*θ*_*r*_ is the change in resonance angle *θ*_*r*_ for Δ*n*_*s*_ change in *n*_*s*_. On the other hand, FoM is defined as [74]

$$\begin{array}{c} \text{FoM}=\frac{S}{\Delta \theta_{i,(1/2)}}, \end{array}$$ (8)

where Δ*θ*_*i*,(1/2)_ is the full-width of *θ*_*i*_ at the half-maximum points on the *R*-profile.

### 3.3 Simulation method

**Fig 4:**
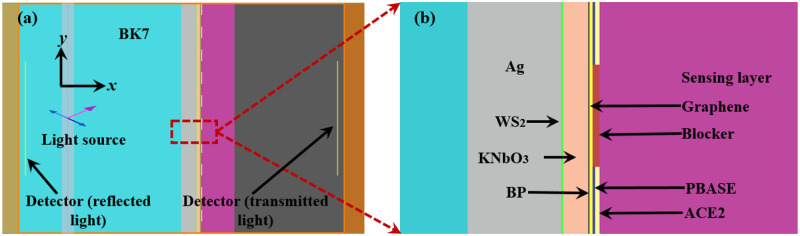
(a) Simulation setup in the *xy* plane and (b) Material layers in the simulation domain.

In this work, we solve 2-D full-field Maxwell’s equations using the FDTD method to calculate the interaction of the incident light with the sensor structure and determine the SPR dynamics. For this purpose, we use Lumerical FDTD Solutions. Since the structure is invariant in the *z*-direction, both 3-D and 2-D models produce the same results [75]. The 2-D simulation setup of the proposed sensor is shown in Fig 4. Fig 4 also shows the positions of the incident light source and reflection and transmission detection planes. The simulation area is 1600 nm in the *x*-direction and 1000 nm in the *y*-direction. We apply a non-uniform meshing technique with ultra-fine mesh grids in FDTD simulations to limit the overall error to <0.05%. Furthermore, the simulation boundaries in the *x*-direction are PML, while Bloch boundaries terminate those in the *y*-direction. The incident light is a plane wave with a 633 nm wavelength and TM polarization. The incident light source is located at 750 nm from the BK7–Ag interface, whereas the reflected light intensity is recorded at 775 nm from the same interface.

For total internal reflection, the incidence angle *θ*_*i*_ must be greater than the critical angle (*θ*_*c*_). The total light absorption of the sensor is *A* = 1 − *T* − *R*, where *T* is the transmission coefficient. Here, as the incident light will experience an attenuated total reflection, *T* = 0; therefore, we can write *A* = 1 − *R*. When SPs are excited at the resonance incident angle, *R* drops sharply. In this work, we vary *θ*_*i*_ from 55° to 85°, with a step size of 0.099° to calculate the *R*-profile for mass level S-protein concentrations. Additionally, for ultra-low-level SARS-CoV-2 detection, we vary *θ*_*i*_ from 65.30° to 65.60°, with a step size of 0.00149°.

## 4 Results and discussion

### 4.1 Detection approach and limit of detection

**Fig 5:**
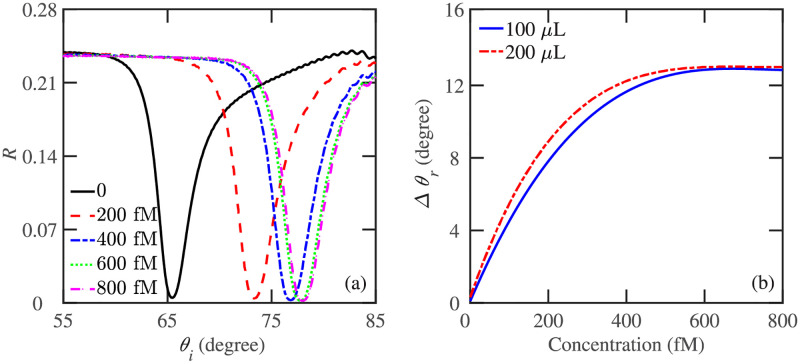
(a) *R*-profile of the proposed graphene SPR sensor for different SARS-CoV-2 S-protein concentrations as a function of *θ*_*i*_. In this case, SARS-CoV-2 S-proteins are added to 100-*μ*L PBS solution. (b) Δ*θ*_*r*_ against SARS-CoV-2 S-protein concentration for 100-*μ*L and 200-*μ*L PBS solution.

As the SARS-CoV-2 S-protein concentration in PBS changes, the sensing layer experiences a refractive index variation. Consequently, the SP wave vector changes, eventually changing *θ*_*r*_. Fig 5(a) shows *R*-profiles of the proposed sensor as a function of *θ*_*i*_ for different SARS-CoV-2 S-protein concentrations. When the sensing layer contains only the PBS, without any SARS-CoV-2 S-protein, *θ*_*r*_ is 65.44°. The change of *R*-profile depends on the refractive indices of the buffer layer, such as the PBS, and the target molecule, such as the S-protein concentration. When the S-protein concentration is 1 fM, *θ*_*r*_ shifts to 65.445° and 65.450° for 100-*μ*L or 200-*μ*L PBS, respectively. Thus, the change in *θ*_*r*_, i.e., Δ*θ*_*r*_, is 0.005° and 0.01° when 1 fM SARS-CoV-2 S-protein is present in 100-*μ*L and 200-*μ*L PBS, respectively, as shown in Fig 5(b). *R*_min_ also decreases slightly when the S-protein concentration increases due to the enhanced absorption of the incident light.

Fig 5(b) shows Δ*θ*_*r*_ = *θ*_*r*(PBS+ACE2+S-protein)_ − *θ*_*r*(PBS+ACE2)_ calculated from the *R*-profiles as the S-protein concentration varies for 100- and 200-*μ*L PBS solutions. We note that Δ*θ*_*r*_ increases significantly with the S-protein concentration. Increasing the S-protein concentration increases the sensing layer refractive index, enhancing light absorption. As a result, the Δ*θ*_*r*_ shifts more. However, Δ*θ*_*r*_ does not vary noticeably when S-protein concentration is ≳ 600 fM in 100-*μ*L PBS solution, as shown in Fig 5(b). Similarly, Δ*θ*_*r*_ shows a saturating behavior when the S-protein concentration is ≳ 500 fM in 200-*μ*L PBS solution. A similar tendency has been observed in the literature, showing Δ*θ*_*r*_ saturation behavior when the S-protein concentration is >104 copies/ml [20]. Now, the refractive index increases more with the S-protein concentration in the 200-*μ*L PBS solution than in the 100-*μ*L PBS solution, as shown in Fig 3. Therefore, the saturation behavior is manifested at a smaller S-protein concentration in the 200-*μ*L PBS solution than in the 100-*μ*L PBS solution. The maximum *θ*_*r*_ shifts are Δ*θ*_*r*(max)_ = 12.56° and 12.72° for 100-*μ*L or 200-*μ*L PBS solutions, respectively.

To detect the SARS-CoV-2 S-protein, Δ*θ*_*r*_ values are used. As S-proteins incrementally adsorb to the sensor surface, *θ*_*r*_ keeps shifting to greater values until it reaches the maximum [76]. S-proteins are detected when Δ*θ*_*r*_ > 0. Now, LoD is determined from the minimum S-protein concentration for which a non-zero Δ*θ*_*r*_ is registered. The proposed sensor shows Δ*θ*_*r*_ > 0 even when the SARS-CoV-2 S-protein concentration is only 1 fM, enabling the proposed sensor to detect as low as 1 fM SARS-CoV-2 S-protein.

The LoD of a sensor is an essential parameter, especially when detecting a critical pathogen like SARS-CoV-2. In Table 2, we compare the LoD of the proposed sensor with some recently proposed sensors that use plasmonic techniques to detect the SARS-CoV-2 S-protein. The proposed sensor shows a much smaller LoD than that reported by these state-of-the-art sensors. We note that the plasmonic meta-sensor of Ref. [19] shows an LoD of ∼4.2 fM, relatively close to that obtained from the proposed sensor in this work. However, the meta-sensor operates in the THz range and is bulky. The meta-sensor also does not work for the entire range of the S-protein concentration.

**Table 2:** Comparisons of LoD of the proposed sensor with different recently proposed sensors for SARS-CoV-2 S-protein detection.

Sensor device	Assay components	LoD
MRT-PCR [77]	SARS-CoV-2	5 *μ*M
dd-PCR [78]	SARS-CoV-2	0.00187 ng
RT-LAMP [79]	SARS-CoV-2	0.025 *μ*g/*μ*l
FET biosensor [20]	SARS-CoV-2	1 fg/ml
Meta-sensor [19]	Gold NPs, SARS-CoV-2	∼4.2 fM
Photo-thermal biosensor [4]	Gold NPs, SARS-CoV-2	0.22 pM
NIR biosensor [3]	ITO, tellurene, MoS_2_-COOH, SARS-CoV-2	∼301.67 nM
LSPR biosensor [80]	Au nano-spikes in opto-microfluidic chip	∼0.5 pM
This work	Graphene, PBASE, ACE2, SARS-CoV-2	1 fM

### 4.2 Sensor resolution and binding affinity

**Fig 6:**
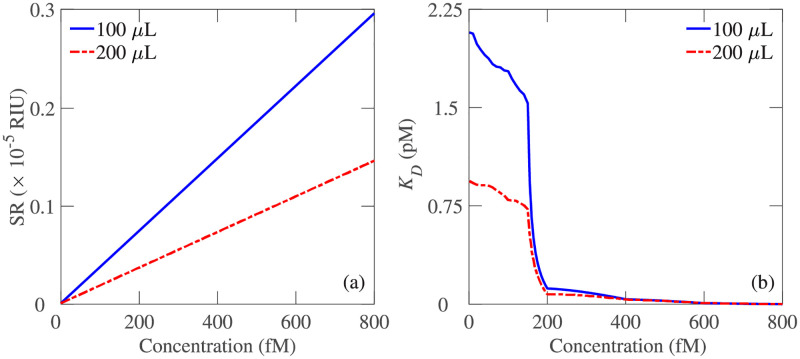
(a) SR and (b) *K*_*D*_ as a function of S-protein concentration of the proposed graphene SPR sensor for 100-*μ*L and 200-*μ*L PBS solutions.

In Fig 6(a), we show the proposed sensor’s resolution (SR) as a function of the S-protein concentration. The resolution of a sensor can be determined by [81]

$$\begin{array}{c} \text{SR}=\Delta n_{s}\;\frac{\Delta \theta_{r(\text{min})}}{\Delta \theta_{r(\text{max})}}, \end{array}$$ (9)

where Δ*θ*_*r*(min)_ is the minimum spectrum resolution, and Δ*θ*_*r*(max)_ is the maximum *θ*_*r*_ shift. The maximum SR is 0.25 × 10^−5^ RIU when the S-protein concentration is 800 fM for 100-*μ*L PBS solution. When the S-protein concentration is 1 fM, the SR is 0.015 × 10^−5^ RIU and 0.016 × 10^−5^ RIU for 100-*μ*L and 200-*μ*L PBS solutions, respectively. The SR of the proposed sensor signifies its ability to detect SARS-CoV-2 S-protein in minute index variations such as on the order of 10^−5^ RIU, which is significant compared to the recently reported SR values in the literature [81–83].

In an SPR sensor, a flow channel is typically used to inject an aqueous solution to the sensor surface [84]. In our proposed sensor, the PBS solution containing SARS-CoV-2 can be injected into the channel where ACE2 antibodies are immobilized on the graphene surface. SARS-CoV-2 S-proteins must bind to ACE2 antibodies on the sensor surface to change the refractive index of the sensing layer. The binding between the immobilized ACE2 antibodies and S-proteins is denoted by the association constant (*K*_*A*_) or the dissociation constant (*K*_*D*_), where *K*_*D*_ = 1/*K*_*A*_. We can derive an expression for *K*_*D*_ using the Langmuir model [16]

$$\begin{array}{c} K_{D}=C[\frac{\Delta \theta_{r(\text{max})}}{\Delta \theta_{r}}-1]. \end{array}$$ (10)

Langmuir model is a ligand binding model to justify the affinity of analyte-antibody bindings [85]. Generally, the *K*_*D*_ value for proteins on an SPR sensor is < 10 nM [86]. The *K*_*D*_ value should be as small as possible because a smaller *K*_*D*_ value represents a greater binding affinity of the sensor to its target element. Fig 6(b) shows *K*_*D*_ of the proposed sensor as a function of the S-protein concentration. We note that the binding affinity between ACE2 and S-protein increases with the S-protein concentration. The *K*_*D*_ value is smaller with the 200-*μ*L PBS solution than that with the 100-*μ*L PBS solution due to the increasing number of S-proteins in greater PBS volume enhancing the chances of binding between ACE2 and S-proteins. We note that the proposed sensor shows a smaller *K*_*D*_ value compared to recent reports on *k*_*D*_ values in the literature [86].

### 4.3 Sensing performance

The selectivity of the proposed graphene SPR SARS-CoV-2 sensor can be determined from the change in *θ*_*r*_ as the S-protein concentration changes [16]. Fig 5(b) shows Δ*θ*_*r*_ for different S-protein concentrations for two PBS solutions. Δ*θ*_*r*_ increases with the S-protein concentration, with the increase more significant for the 200-*μ*L PBS solution, signifying that the proposed sensor has an affinity toward S-proteins [48]. As Δ*θ*_*r*_ sensitively changes with the S-protein concentration, the proposed sensor is highly selective of the S-protein. The proposed sensor shows comparatively greater selectivity compared to recent reports in the literature [16, 87, 88].

**Fig 7:**
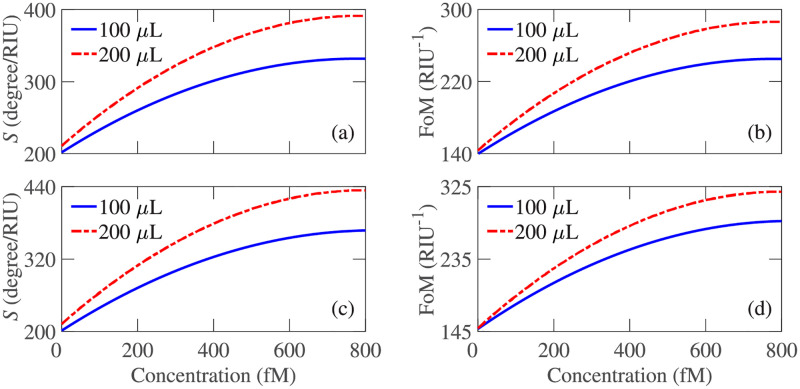
(a) *S* and (b) FoM using BP and (c) *S* and (d) FoM using BlueP as a function of S-protein concentration of the proposed graphene SPR sensor for 100-*μ*L and 200-*μ*L PBS solutions.

Fig 7(a) shows the sensitivity of the proposed graphene SPR sensor as a function of the SARS-CoV-2 S-protein concentration. As the sensitivity depends on the change of *θ*_*r*_ and increasing S-protein concentration shows a more significant change in *θ*_*r*_, we find that the sensitivity increases as the S-protein concentration increases. Also, the 200-*μ*L PBS solution shows greater sensitivity than the 100-*μ*L PBS solution because the S-protein number increases with PBS solution volume, which increases Δ*θ*_*r*_. When the S-protein concentration is 1 fM, *S* = 201 degrees/RIU and 210 degrees/RIU for 100-*μ*L and 200-*μ*L PBS solutions, respectively. Furthermore, the proposed sensor shows the maximum sensitivity of 371 degrees/RIU when the S-protein concentration is 800 fM for 200-*μ*L PBS solution.

The FoM of the proposed sensor has been presented in Fig 7(b) as a function of the S-protein concentration. We note that FoM increases with the increase of S-protein concentration. When the S-protein concentration is 800 fM, the FoM is maximum with values of 233 RIU^−1^ and 275 RIU^−1^ for 100-*μ*L and 200-*μ*L PBS solutions, respectively. Moreover, 1 fM S-protein concentration shows 140 RIU^−1^ and 148 RIU^−1^ FoM for 100-*μ*L and 200-*μ*L PBS solutions, respectively. As the increasing S-protein concentration raises *n*_*s*_, *θ*_*r*_ increases. In addition, as the FoM is directly related to the sensitivity according to Eq (11), it increases when the sensitivity increases.

Fig 7(c) shows the sensitivity of the proposed sensor as a function of the SARS-CoV-2 S-protein concentration using BlueP instead of BP. The sensitivity increases as the S-protein concentration increases. The sensitivity with BlueP is comparable to that with BP at the ultra-low-level S-protein concentration. However, when the S-protein concentration increases, the sensor with BlueP shows greater sensitivity than with BP. When the S-protein concentration is 800 fM, the sensitivity for the sensor with BlueP is 365 degrees/RIU and 435 degrees/RIU for 100-*μ*L and 200-*μ*L PBS solutions, respectively, ∼10% greater than the sensor with BP.

Fig 7(d) shows the FoM as a function of the SARS-CoV-2 S-protein concentration using BlueP instead of BP. BlueP shows greater FoM than BP for ultra-low-level and mass-level S-protein concentrations. For example, when the S-protein concentration is 1 fM, FoMs for the sensor with BP are 152 RIU^−1^ and 153 RIU^−1^ for 100-*μ*L and 200-*μ*L PBS solutions, respectively, ∼4% greater than the sensor with BP. Furthermore, when the S-protein concentration is 800 fM, FoMs for the sensor with BlueP are 282 RIU^−1^ and 318 RIU^−1^ for 100-*μ*L and 200-*μ*L PBS solutions, respectively, ∼15% greater than the sensor with BP. The enhancement in FoM with the BlueP can be attributed to its narrower *R* spectrum than BP.

**Fig 8:**
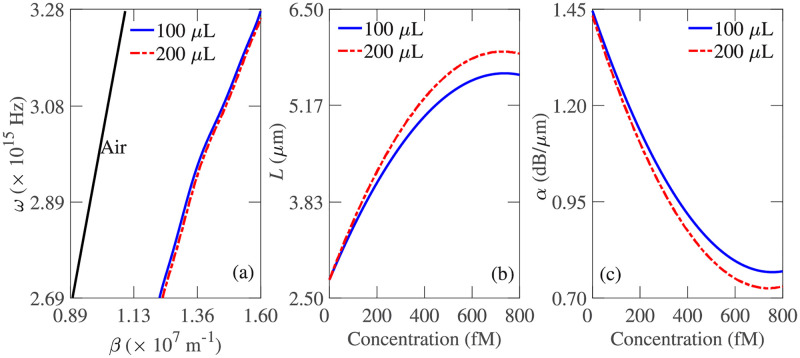
(a) Dispersion relations of the proposed sensor when the S-protein concentration is 1 fM using BP. The most left straight line is the light line, (b) Propagation length, and (c) Propagation loss of the proposed sensor as a function of the SARS-CoV-2 S-protein concentration for 100-*μ*L and 200-*μ*L PBS solutions.

As the proposed sensor has several layers of different refractive indices, calculating the dispersion relation is numerically challenging. This work analyzes the dispersion relations using the radiative mode [89]. The in-plane wave vector can be given by [89]

$$\begin{array}{c} \beta =\frac{\omega \sqrt{\varepsilon_{\text{prism}}}}{c}\text{sin}\theta_{r}, \end{array}$$ (11)

where *ε*_prism_ is the dielectric constant of the prism, *ω* is the angular frequency, and *c* is the speed of light in vacuum. Fig 8(a) shows the dispersion relation of the proposed sensor. We compute the reflectivity of the proposed sensor as a function of the frequency and *θ*_*i*_. The frequency is varied from 4.2827 × 10^14^ Hz to 5.2138 × 10^14^ Hz and *θ*_*i*_ is varied from 55° to 85°. We find that the dispersion curve moves away from the air light-line. We note that at >630 nm wavelength (*ω* = 2.9 × 10^15^ Hz), dispersion relations for both 100-*μ*L and 200-*μ*L PBS solutions move farther from the light line.

On the other hand, SPPs suffer damping in metal, decreasing the propagation length significantly [90]. Mainly, damping depends on the dielectric constant of metal at the oscillation frequency of SPPs. Losses may also occur due to the coupling of SPPs to radiation modes. Propagation loss depends on the sensing layer’s dielectric constant [91]. The propagation length can be defined by [92]

$$\begin{array}{c} L=\frac{1}{\Delta \theta_{i,(1/2)}n_{p}\frac{\omega }{c}cos\theta_{r}}, \end{array}$$ (12)

where *n*_*p*_ is the refractive index of the prism. Also, the propagation loss can be calculated by [92]

$$\begin{array}{c} \alpha =[-10\text{log}(1/e)]/L\approx 4.343/L. \end{array}$$ (13)

Fig 8(b) shows that the propagation length increases with the SARS-CoV-2 S-protein concentration. Also, the 200-*μ*m PBS solution shows a greater propagation length than the 100-*μ*m PBS solution since the sensing layer’s refractive index increases with the S-protein concentration, enhancing light confinement. Our proposed sensor shows a small propagation length <10 *μ*m, which is a short-range SPP. As a result, the propagation loss decreases with the S-protein concentration, as shown in Fig 8(c). When the propagation length increases from 2.62 *μ*m to 5.84 *μ*m, the propagation loss decreases from 1.44 dB/*μ*m to 0.72 dB/*μ*m for 200-*μ*m PBS solution. Compared to the existing literature, the proposed sensor performs better in decreasing the loss [92].

The proposed sensor’s sensitivity and FoM performances are significantly better than the state-of-the-art optical sensors. We compare the sensitivity and FoM of the proposed sensor in Table 3 with some recently reported sensors. We have compared the minimum sensitivity and FoM achievable from the proposed sensor at the LoD with those reported in the literature. We have considered the sensors for comparison that specifically use similar 2-D materials. The compared sensors also operate at 633 nm incident wavelength and report results for a sample of ∼1.3349 refractive index, which is the same *n*_*s*_ value of this work at 1 fM S-protein concentration in 200-*μ*L PBS solution.

**Table 3:** Performance comparison of our proposed sensor with different recently proposed sensors.

Sensor configuration	*S* (degrees/RIU)	FoM (RIU^−1^)
Prism /TiO_2_ /SiO_2_ /Ag /MoS_2_ /Graphene /Sensing layer [93]	98	88.89
Prism /ZnO /Ag /BaTiO_3_ /Graphene /Sensing layer [94]	157	71
Prism /Au /PtSe_2_ /Graphene /Sensing layer [95]	154	–
Prism /Ag /PtSe_2_ /Sensing layer [96]	162	15
Prism /Al /Au /Graphene /WSe_2_ /Sensing layer [97]	164	–
This work (BP/BlueP)	210/210	148/153

## 5 Conclusion

Tackling the COVID-19 pandemic requires rapid, low-cost, and sensitive detection of ultra-low-level SARS-CoV-2. The proposed sensor will detect SARS-CoV-2 in real time without requiring any label or complicated sample preparation. The proposed sensor shows a significant change in the SPR resonance when the SARS-CoV-2 concentration varies even at the femtomolar level, and hence is suitable for sensitive ultra-low-level SARS-CoV-2 detection and early detection of COVID-19. The proposed sensor’s sensitivity and FoM performances are significantly better than the state-of-the-art optical sensors. The proposed sensor also shows much stronger binding kinetics with the sensor surface than recent reports in the literature. The results and analysis confirm that the proposed sensor is promising for SARS-CoV-2 detection and may find applications in detecting other biochemical and biological analytes.
